# Changing case definition: An analysis of its impact on Lyme disease surveillance in Nova Scotia

**DOI:** 10.14745/ccdr.v52i0102a06

**Published:** 2026-02-19

**Authors:** Kelachi Nsitem, Jennifer Cram, Aini Khan, Colleen Ryan, Todd Hatchette, Shelley Deeks, Linda Passerini, Molly Trecker, Kathryn McIsaac

**Affiliations:** 1 Department of Health and Wellness, Government of Nova Scotia, Halifax, NS; 2 Department of Pathology and Laboratory Medicine, Nova Scotia Health, Halifax, NS; 3 Department of Pathology, Dalhousie University, Halifax, NS; 4 Department of Community Health and Epidemiology, Dalhousie University, Halifax, NS

**Keywords:** Lyme disease, tick-borne disease, surveillance, Nova Scotia

## Abstract

**Background:**

Nova Scotia has experienced a growing number of Lyme disease (LD) cases since 2002. From 2009 to 2022, Nova Scotia adopted a LD case definition that aligned with the Public Health Agency of Canada’s definition. On January 1, 2023, Nova Scotia transitioned to a LD definition that relies on laboratory evidence alone.

**Objectives:**

To describe and compare historic trends in confirmed LD case counts and incidence under the former and current LD case definitions between 2018 and 2023 and assess the impact of the case definition change on LD surveillance.

**Methods:**

Confirmed LD cases were extracted from Nova Scotia’s Electronic Public Health Information System, software Panorama, according to the former case definition for the years 2018–2022 and the current case definition for the years 2019–2023. As the 2018 laboratory data in Panorama was incomplete, raw data for 2018 were obtained from Nova Scotia’s Provincial Public Health Laboratory Network. Confirmed case counts and incidence rates per 100,000 population were calculated by year, sex, age group and geographic zone, under both case definitions. Seasonality was determined by the reporting date of the case.

**Results:**

From 2018–2022, the current case definition identified 4,238 cases, a substantial increase of 2,493 cases over the 1,745 reported by the former case definition, with an additional 2,058 cases in 2023 under the current case definition. This led to a clear upward trend in confirmed incidence rates with the current case definition, unlike the variable pattern seen with the former case definition. Males and individuals aged 5–14, 40–59, and ≥60 years experienced consistently higher sex and age-specific rates under both case definitions. The Western Zone consistently reported the highest incidence rates. Seasonally, both case definitions showed reporting peaks from June to September, with the peak occurring two-week later peak with the current case definition.

**Conclusion:**

When the current LD case definition was applied to historic surveillance data, past rates of confirmed LD increased suggesting under-reporting of clinical presentation of LD to public health in high incidence jurisdictions.

## Introduction

Lyme disease (LD), the most reported tick-borne disease in North America, is transmitted to humans through the bite of a tick infected with *Borrelia burgdorferi* ((1–4)). In Nova Scotia, the blacklegged tick, *Ixodes scapularis*, carries this bacterium ((5)). The disease typically manifests with erythema migrans (EM), in approximately 80% of patients who are infected, often accompanied by other early disease symptoms such as fatigue or fever ((6–8)). If left untreated, dissemination of the disease may result in multiple EM lesions, along with cardiovascular (temporary atrioventricular blocks), musculoskeletal (joint swelling or arthritis) and neurologic manifestations (facial palsy, neuropathy or encephalopathy) ((3,7,8)).

Nova Scotia has experienced a growing number of LD cases since 2002, the Department of Health and Wellness declared the entire province of Nova Scotia an “at risk area” in 2017, defined as a location with evidence of reproducing populations of known tick vectors and the likely transmission of *B. burgdorferi* ((9)). In 2022, the reported incidence (confirmed and probable) of LD in Nova Scotia was almost five times greater than the national incidence ((4)). Furthermore, incidence of LD is projected to increase because climate change is likely to expand the abundance and distribution of tick populations ((10)).

Lyme disease became a nationally notifiable disease in 2009 ((4)). From 2009 through 2022, Nova Scotia adopted a LD case definition (CD) that aligned with the Public Health Agency of Canada’s CD ((11)). This CD required both clinical information and laboratory evidence for a confirmatory case, and EM was captured within the probable CD ((11)). In 2023, Nova Scotia transitioned to a new CD, relying on laboratory evidence alone for confirmed cases ((12)). This approach aligns with the 2022 revised high-incidence LD CD by the Centers for Disease Control and Prevention in the United States ((13)). This change means that confirmed cases rely solely on laboratory evidence. Laboratory testing is not recommended in early stage LD (i.e., localized EM) due to its poor sensitivity ((8,14)). As such, public health will not be notified of EM with the current CD and these cases will not be included in Nova Scotia’s confirmed or probable LD case counts.

Coupled with the increase in LD cases, evidence suggests that the burden of submitting clinical information for both probable and confirmed cases can result in under-reporting of LD ((15)). Additionally, during the COVID-19 pandemic, competing public health priorities resulted in reduced capacity for LD investigation by public health within the province ((2,13,16)).

The objective of this surveillance report is to describe and compare historic trends in confirmed LD counts and incidence over the period from 2018 to 2023, and to assess the impact of the CD change on LD trends.

## Methods

### Setting and population

This report includes all confirmed LD cases reported to public health in Nova Scotia from January 1, 2018, to December 31, 2023. Lyme disease is a notifiable disease in Nova Scotia, and any cases that met either confirmatory or probable CDs are to be reported and captured in the province’s Public Health Information System, Panorama. The year 2018 was selected to align with the year that Panorama was implemented.

### Case definitions and detection

Between January 1, 2018 and December 31, 2022, a confirmatory case of LD required both clinical evidence and laboratory confirmation ((11)). Probable cases required EM rash (determined by clinical presentation without laboratory tests) or clinical evidence of illness with laboratory evidence of infection, without history of residence in or visit to a LD risk area (national case definition) ((11)). As of January 1, 2023, a confirmed case of LD must have confirmatory laboratory evidence; however, clinical evidence is no longer required ((17)). Individuals presenting with localized EM will not receive laboratory testing given the high likelihood of a false negative serology test in early stage LD ((8,14)).

Probable cases are those with only presumptive laboratory evidence (i.e., positive IgG immunoblot); EM (i.e., clinical criteria) is no longer captured in the probable CD (Nova Scotia case definition). Full CDs are in the **Appendix**, as Supplemental material (Table S1).

On April 1, 2021, the modified two-tier test (MTTT) for serologic testing of LD was introduced in Nova Scotia, replacing the standard two-tier test (STTT). The MTTT has approximately 25% greater sensitivity in the detection of early LD ((11,12,14,18)). Both STTT and MTTT are included as confirmatory laboratory evidence of infection in the CD.

### Data sources

Reported LD cases were obtained from Panorama. All reported cases meeting the Nova Scotia LD CD between 2018 and 2022 were obtained using investigations in Panorama. Investigations are conducted by public health nurses and include clinical evidence from physicians as well as additional demographic, risk factor and geographic data. To retrospectively apply the current CD, we assessed the stand-alone laboratory results (i.e., those with no associated case investigation ID attached). These were extracted directly from Panorama (2019–2023) and from raw data from Nova Scotia’s Provincial Public Health Laboratory Network for 2018. The current CD was applied to the data to determine the number of cases meeting the new CD. Population characteristics were extracted from Statistics Canada’s annual (July 1) population estimates for each year (2018–2023). The Statistics Canada annual population estimate corresponded with the year the case occurred.

### Analysis

Confirmed case counts and incidence rates per 100,000 population were calculated by year, sex and age group under the former and current Nova Scotia CD using confirmed cases as the numerator and the census population estimates from Statistics Canada data for 2018–2023 as the denominator ((19)). The current confirmed LD CD was applied to analyze geographic trends using the client’s active address at the time of specimen collection. Four geographic areas were used to correspond to Nova Scotia administrative health zones (Northern, Eastern, Central, Western). For 2018, the raw data included age at the time of data extraction and date of specimen collection but did not include age at diagnosis, date of birth or geography. Age at diagnosis was determined by manually extracting and reviewing the records in Panorama or the Provincial Public Health Laboratory Network. If age at diagnosis was still unavailable, it was estimated by applying the average age difference between age at data extraction and specimen collection date. Age groups were selected to align with Nova Scotia provincial Notifiable Disease reporting and were collapsed in accordance with incidence trends provincially and nationally. Seasonality was determined by the case reporting date, which, under the former CD, could be the first clinical diagnosis, symptom onset, or laboratory collection date; under the current CD, it is the laboratory collection date.

We performed a sensitivity analysis to account for the increased number of cases expected with the new MTTT methodology compared to the STTT. This analysis assumed the MTTT was not introduced and involved applying a 25% decreased sensitivity to incident cases after 2020.

## Results

Lyme disease trends over time under the former and current Nova Scotia confirmed case definition

**Figure 1:**
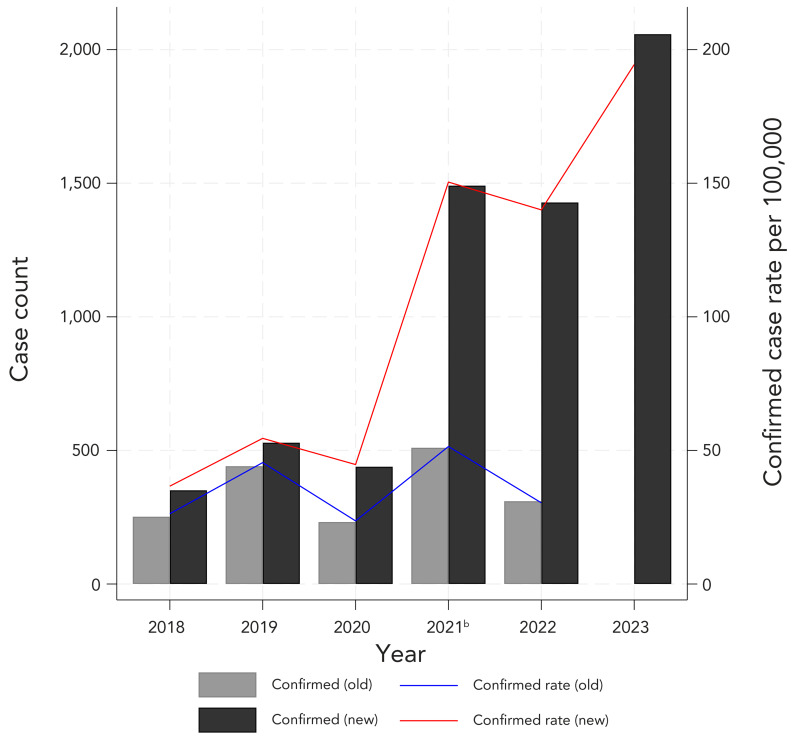
Reported confirmed Lyme disease counts and incidence rate^a^, with the former and current definitions, 2018–2023^b^ ^a^ Denominators for yearly rates per 100,000 population were obtained from Statistics Canada, population estimates on July 1 ^b^ April 1, 2021 modified two-tier test introduced, replacing the standard two-tier test

**Figure 1** shows the confirmed case counts and incidence rate of LD over time using the former and current CD. Nova Scotia reported 1,745 confirmed cases of LD between 2018 and 2022 using the former CD. After retrospectively applying the current CD, a total of 4,238 cases would have met CD between 2018 and 2022 (+2,493). An additional 2,058 confirmed cases were reported in 2023 under the current CD. Confirmed case counts were higher each year when the current CD was retrospectively applied. Moreover, the confirmed incidence rates over time exhibited a clear upward trend with the current CD whereas the confirmed case rates showed year to year variability and no clear directional trend with the former CD. In 2018, the percent difference in confirmed LD cases between CD was 33%, dropping to 18% in 2019, but rising sharply from 62% in 2020 to 129% in 2022.

### Demographic characteristics of confirmed cases

**Figure 2:**
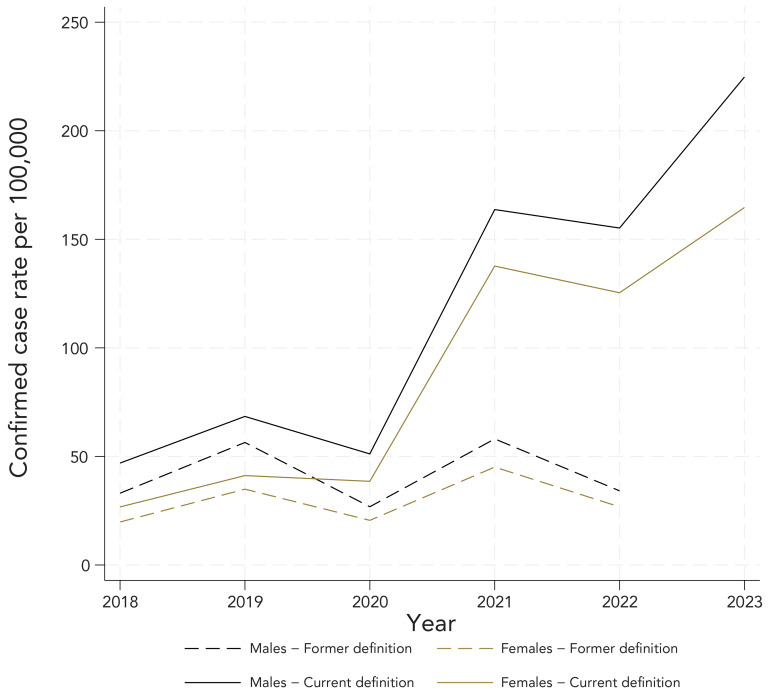
Confirmed incidence rate^a^ of reported Lyme disease cases by sex, with the former and current case definition, 2018–2023 ^a^ Denominators for yearly rates per 100,000 population were obtained from Statistics Canada, population estimates on July 1

**Figure 3:**
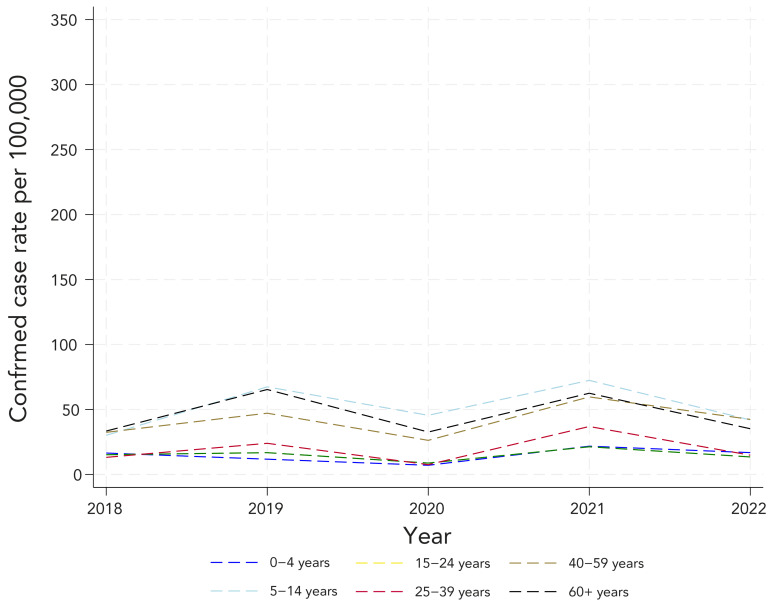
Confirmed incidence rate^a^ of reported Lyme disease cases by age group, with the former definition, 2018–2022 ^a^ Denominators for yearly rates per 100,000 population were obtained from Statistics Canada, population estimates on July 1

**Figure 4:**
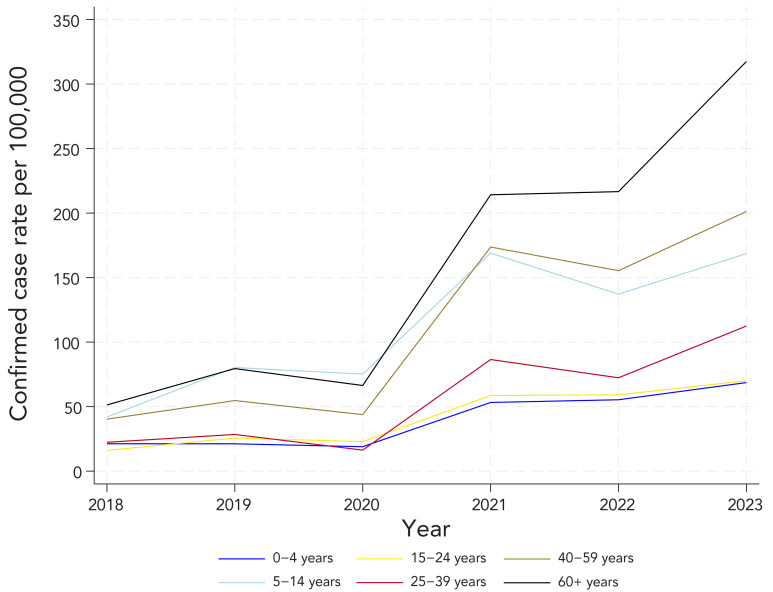
Confirmed incidence rate^a^ of reported Lyme disease cases by age group, with the current definition, 2018–2023 ^a^ Denominators for yearly rates per 100,000 population were obtained from Statistics Canada, population estimates on July 1 of each year (2018–2023)

Complete demographic information was available for all reported cases of LD obtained from the investigations (former CD) and 54% of age-data from 2018 laboratory records had to be estimated (current CD). **Figure 2** presents sex-specific rates. Males experienced higher sex-specific incidence rates of confirmed LD compared with females. The sex-specific trends were similar to the overall population trends. **Figure 3** and **Figure 4** show age-specific incidence rates over time in the former and current CD, respectively. Cases ranged in age from birth to 97 years. Individuals aged 5–14 years, 40–59 years and 60 years and older consistently experienced higher age-specific rates of confirmed LD across all years under both CD.

### Geographic distribution of confirmed cases

**Figure 5:**
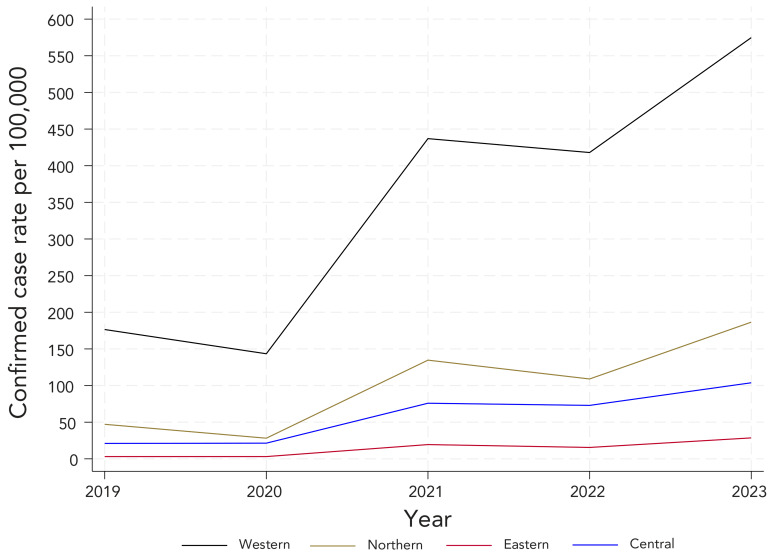
Confirmed incidence rate^a^ of reported Lyme disease cases by zone, with the current case definition, 2019–2023 ^a^ Denominators for yearly rates per 100,000 population were obtained from Statistics Canada, population estimates on July 1

Information on location was available for 5,886 (98.8%) cases under the current CD, from 2019 onwards and was unavailable from 2018 laboratory data. **Figure 5** displays the incidence rate of LD by zone using the current CD. The incidence rate was consistently highest in Western Zone. Under the former CD, most Nova Scotia confirmed cases were in the Western Zone in 2018 to 2020. In 2021 and 2022, Western Zone made up 15% or less of all cases in Nova Scotia. When applying the current CD, the majority of cases were in Western Zone in all years (Figure S1).

### Seasonal distribution

**Figure 6:**
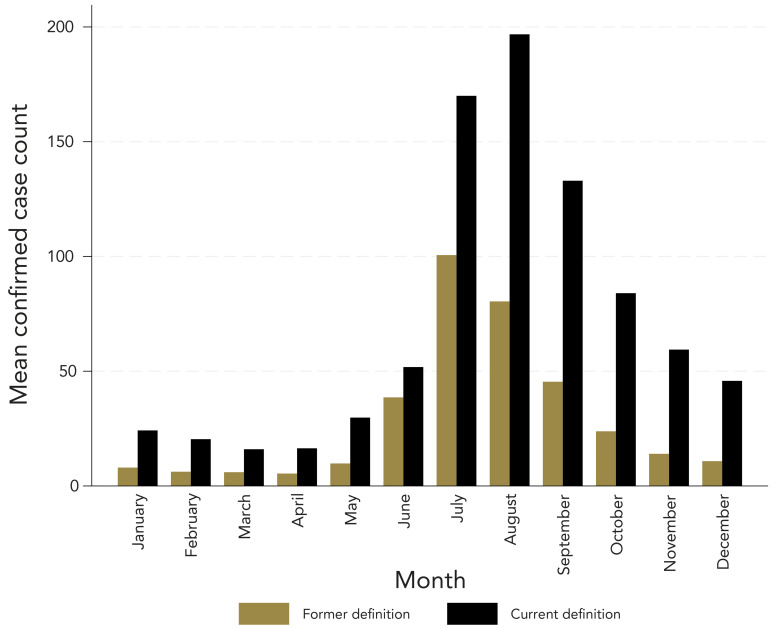
Mean number of confirmed Lyme disease cases by month, based on reporting date^a^, with the former and current case definitions, 2018–2022 ^a^ The date of the first clinical diagnosis, symptom onset, or the laboratory collection date for the former Lyme disea definition OR the laboratory collection date for the current Lyme disease definition

**Figure 6** shows the seasonality of confirmed cases of LD. Reporting dates peaked from June to September with both LD CDs. July was the peak month under the former LD CD, while August was the peak month under the current LD CD. On average, the peak reporting date with the current LD CD occurred two weeks later than with the former CD.

### Impact of serologic testing on Lyme disease trends

An increase in serologic testing volume for LD over time was observed using crude laboratory LD test volume counts from the Provincial Public Health Laboratory Network. The trend in testing volume mirrored the trend in reported case counts with the current LD CD (Figure S2).

After applying a sensitivity analysis to account for the introduction of MTTT in 2021, a similar trend of increased confirmed cases over time remained with lower year specific rates (Figure S3 and Figure S4).

## Discussion

### Comparison of trends with the former and current Lyme disease case definitions

To optimize public health resources and strengthen surveillance, surveillance systems should be periodically evaluated, focusing on attributes such as data quality, acceptability and positive predicative value ((20)).

Applying the current Nova Scotia LD CD retrospectively to reported LD cases from 2018–2022 increased the number of reported confirmed cases from 1,745 to 4,238. The key distinction in the current CD is the removal of the requirement for clinical evidence to confirm a case. Clinical evidence may have been a barrier to submitting LD case data, resulting in under-reporting. This could be particularly true when the number of cases is high, as we would expect in a high-incidence jurisdiction.

There has been discourse around the potential underreporting of LD cases in human surveillance, highlighted in literature from Canada and the United States ((15,21)). While more research is needed to understand possible reasons for under-reporting, clinicians may not be reporting clinical symptoms because of the additional time burden, or they fail to see value in reporting since clinical evidence does not provide information that could be used to prevent cases of LD ((6,13)). This may be the result of low acceptability of the former CD.

It is also possible that a true absence of symptoms that meet CD, rather than under-reporting of symptoms, explains some of the difference between the number of cases in the former and current CD. If symptoms were not present, there is a greater likelihood of including false positives with the current CD. Nonetheless, while clinical information improves LD pre-test probability and influences the positive predictive value, in a high incidence jurisdiction, the proportion of people who test positive who are truly positive is higher than a low incidence jurisdiction. This is supported by two LD studies showing LD seropositivity rates in Nova Scotia increasing from 1% to 1.6% from 2012 to 2023 ((22,23)).

The former and current LD CDs showed similar trends for both sex-specific and age-specific rates of LD. Males experienced higher rates of confirmed LD and those aged 5–14 years, 40–59 years and 60 years and older experienced the highest age-specific rates of LD. These demographic trends mirror historical patterns of LD distribution in Nova Scotia. The Western Zone experienced the highest zone-specific rates of confirmed LD, followed by the Northern Zone. This also follows environmental and historical trends in LD risk areas, where South-Western Nova Scotia was the earliest LD endemic area ((5)). Before the change LD CD, varying workload demands and clinician reported behaviour may have resulted in inconsistent reporting methods between zones. As a result, geographic trends based on the former CD may not accurately reflect the burden of disease within the zones and were therefore omitted from the main geographic analysis.

When considering both LD CD, cases peaked from late spring until the end of summer, with the highest number of cases falling within the months of June, July, and August. The former CD required clinical information to be collected, resulting in reporting dates encompassing date of symptom onset or clinical diagnosis. The time lag in reporting date, when applying the current CD likely reflects the expected lag time between symptom onset and presenting for assessment and testing contributing to the later reporting dates ((13)).

### Additional factors influencing Lyme disease case trends

Other factors that may have contributed to the LD trends observed include 1) the COVID-19 pandemic, 2) climate change and 3) increased awareness and testing.

The COVID-19 pandemic led to the disruption in public health follow-up on positive laboratory tests, which resulted in an overall decrease in reported LD cases in 2020. Further, a sustained greater reduction in reported cases in 2021 and 2022, relative to laboratory-confirmed cases under the current CD, may reflect reduced public health capacity during those years. Following the pandemic, public health follow-up for LD never fully returned to its pre-pandemic level. Behaviour changes, such as time spent outdoors, may have been influenced by the pandemic, possibly impacting LD cases. Further, the inability to access primary care or delay in primary care delivery may have decreased the number of LD cases diagnosed and reported ((24,25)). The exact magnitude and direction of these effects are unclear.

The consequences of climate change, including increasing temperatures, may contribute to the expansion of habitat and host populations for infected ticks and to increased outdoor human activity—increasing tick abundance and potential for LD transmission ((4,10,26)). In Nova Scotia, compared to the 30 year climate stable period from 1961 to 1990, the subsequent 30 years from 1990 to 2020 have seen a statistically significant increase in the overall mean temperature across all months of the year ((27)). Additionally, there has been a decline in the number of frost days in both spring and autumn, with frost ending earlier in the spring and starting later in the autumn, resulting in an increase in number of days that ticks could be active ((27)).

As the number of LD cases has increased, so has both clinical and public awareness, potentially contributing to increased health-seeking behaviours and clinician suspicion for LD ((4)). An increase in serologic testing volume for LD over time was observed using crude laboratory LD test volume counts from the Provincial Public Health Laboratory Network. An increase in test volume provides the opportunity for more frequent detection and reporting of LD.

Introduced April 1, 2021, the MTTT has shown to be approximately 25% more sensitive for detection of early LD with equivalent specificity to the STTT ((14,18)); however, sensitivity to early stages of LD is estimated to still be relatively low, at approximately 70% ((18)). It is likely that the increase in cases observed after April 1, 2021, could be partly attributed to increased sensitivity in detecting earlier cases of LD, thus capturing cases in people with early infection. Although healthcare workers are encouraged to treat cases of EM without testing for LD, not all patients with early infection present with an EM rash, leading to testing for atypical presentations.

Like the STTT, the MTTT is unable to distinguish between active and past LD infections as the antibody response to the bacteria may persist for years after initial infection; therefore, a positive laboratory result may reflect previous rather than current infection. The impact of this may increase over time as the prevalence of LD increases ((14)).

### Limitations

Crude laboratory data from 2018 were used, applying the current LD CD retrospectively. Thus, a positive enzyme immunoassay and subsequent positive IgG or IgM, were considered as confirmatory, regardless of negative IgM or IgG results, in line with the current Nova Scotia LD CD. Age data for 54% of individuals in 2018 were approximated; however, given the broad age categories, this likely had minimal impact on observed trends. Furthermore, published 2022 population estimates were applied to 2018–2022 and the 2023 geographic zone analysis; at the time of analysis, Statistics Canada 2023 zone population estimates were not available. Since population changes from 2022 to 2023 were minimal, the impact on the calculated rates in this analysis are likely minor.

## Conclusion

After retrospectively applying Nova Scotia’s current CD to historic surveillance data, there was a clear increase in the number of cases of LD identified and an upward trend in LD. While the overall upward trend observed with the current LD CD aligns with historical patterns of LD expansion in Nova Scotia (2009–2018), the change in CD inherently limits direct numerical comparisons with data collected under the former definition or with other jurisdictions using different criteria.

Relying on a laboratory-based CD strengthens the surveillance system’s ability to meet system goals and monitor the burden of LD and trends in LD in the province. Moreover, using only laboratory evidence to monitor LD reduces the burden of investigations on front-line public health staff, freeing resources that can be redirected towards LD awareness campaigns to prevent tick bites and promote appropriate LD management.

Nova Scotia has worked with provincial and federal partners on knowledge translation efforts. Effective communication strategies, including data notes in surveillance reports and engagement with healthcare providers, are crucial to explain the reasons behind the observed increase in reported cases and prevent misinterpretation of LD trends.

Under-reporting is a substantive limitation in high incidence jurisdictions, like Nova Scotia, and the laboratory-based approach improves the accuracy, timeliness, flexibility and acceptability of LD surveillance.

## Appendix

Supplemental material is available upon request to the author: surveillancedhw@novascotia.ca

Table S1: Lyme disease case definitions

Figure S1: Confirmed incidence rate of reported Lyme disease cases by zone, with the former case definition, 2018–2022

Figure S2: Nova Scotia Lyme disease serology testing volume and rate, 2018–2023

Figure S3: Sensitivity analysis of reported confirmed Lyme disease counts and incidence rate, with the former definition, 2018–2022

Figure S4: Sensitivity analysis of reported confirmed Lyme disease counts and incidence rate, with the current definition, from 2018–2023
